# Micro-Coevolution of Genetics Rather Than Diet With Enterotype in Pigs

**DOI:** 10.3389/fnut.2022.846974

**Published:** 2022-03-22

**Authors:** Ning Ma, Yiwei Sun, Jiashun Chen, Zengkai Qi, Chunchen Liu, Xi Ma

**Affiliations:** ^1^State Key Laboratory of Animal Nutrition, College of Animal Science and Technology, China Agricultural University, Beijing, China; ^2^College of Animal Science and Technology, Hunan Agricultural University, Changsha, China

**Keywords:** enterotype, pig, diet, genetic, microbe, micro-coevolution

## Abstract

Based on the characteristic of low diarrhea in native Chinese breeds, we introduce the enterotype model for piglets, which is a new perspective to decipher the colonization and the transition of the gut microbiota among various pig breeds. After eliminating environmental influences represented by diet, the microbiota, mainly shaped by host genetics, is focused. Three representative enterotype clusters were identified, which were represented by *Bacteroides, Streptococcus*, and *Lactobacillus*. Chinese native breeds were distributed in enterotype 1 (E1) and E3, which collectively drove the diversification and functionality of the microbial community of various Chinese pig breeds. Next, the *Lactobacillus reuteri* (*L. reuteri*), which is the representative strain of E3, was specifically isolated in all three enterotypes. The excellent stress-resistance of *L. reuteri-E3* not only highlighted the stronger disease resistance of Chinese breeds but also had a great potential to intervene in weaned piglet diseases. Enterotype classification based on host genetics is much more deterministic and predictable, clarifying the driver of the host-microbiome dynamics and constructing the picture of the micro-coevolution of human host genetics with the gut microbiome.

## Introduction

The diarrhea of weaned piglets is the biggest challenge to the development of intensive pig farming. Fortunately, some native Chinese breeds show a stronger disease resistance in practical production (1, 2), accompanying the development of gastrointestinal mucosal structure and discrepant gut microbiota. Gut microbial communities are involved in a series of regulations, from nutrition to disease defense (3, 4). Understanding the essential drivers of host-microbiome dynamics, and ultimately clarifying biomarkers that efficiently regulate intestinal health, could provide excellent opportunities to construct associations between stable microbiota and diseases, and improve the weaker anti-stress capacity of commercial hybrid pigs to reduce the use of antibiotics (5, 6).

An especially diverse bacteria ecosystem plays an important role in the gut, impacting a series of pathologies in diverse ways (7). Taxonomic and functional differentiation also occurs in both population and individual levels of microbiota (8, 9), and ultimately shapes some core species that are generally shared among different individuals and contribute most to gut microenvironment homeostasis. Nonetheless, questions related to the nature and origin of the intestinal flora community remain to be clarified. In order to find core clusters with stable, deterministic and predictable characteristics, enterotype was identified. Enterotype is distinct clusters characterized by the abundance of signature bacterial genera, which are characterized as “densely populated areas in a multidimensional space of community composition” (10). The occurrence of enterotype (E1, *Bacteroides*; E2, *Prevotella*; and E3, *Ruminococcus*) was first proposed in diverse human populations (10, 11), then, mirrored in chimpanzees (12), and have been introduced to bumblebees (3), wild mice (13), and african buffaloes (14). However, the classification of enterotypes of diverse pig breeds is still lacking.

Although the gut microbiota of commercial hybrid pigs have been extensively explored, the connection between native pig breeds and their flora are less understood. Abundant Chinese native pig breeds, along with differences in disease resistance, will be an important resource for mapping pig functional enterotypes. Characterizing the gut microbiota diversity in various Chinese pig breeds, not only contributes to the exploration of microbiota variation on a larger geographic scale, but also has the potential to identify drivers of host-microbiota dynamics to maximize agricultural productivity.

However, the enterotype paradigm has not been thoroughly explored. Its fragile plasticity is disturbed by environmental factors such as geographical location and dietary factors (14). To relatively maximize the control of environmental effects, four types of native Chinese breeds, which include Tibet pigs (Tibet), Beijing Black pigs (BeiJH), Bama pigs (BaM), and Ningxiang pigs (NingX), are removed to the same place with commercial hybrid Duroc-landrace-Yorkshire piglets (Con), while feeding the same diet. Therefore, in this cohort, a non-genetic heterogeneity is relatively well-controlled, while the genetic distinction is dominant, to achieve a deterministic and predictable enterotype division.

## Materials and Methods

The detailed experimental procedures were described in the method section in Supplementary Material.

### Sample Collection

Fecal samples were collected following a standardized procedure (Supplementary Method). To relatively maximize the control of environmental effects, four types of native Chinese breeds, which include Tibet, BeiJH, BaM, and NingX, were removed from the same place with a commercial hybrid Con, while feeding with the same diet. At the same time, to ensure the stability of the microbial structure, piglets after weaning were selected in this experiment, and the age of the experimental subjects remained the same.

### Bacteria

An appropriate amount of fresh pig feces was added to the modified Man Rogosa Sharpe Medium (MRS) liquid medium, and after 24 h of enrichment culture, the medium was streaked on the modified MRS agar plate with an inoculation loop, and the anaerobic culture was carried out at 37°C for 24–48 h. Colonies of different colors were streaked and inoculated, followed by pure culture. Microscopic observation and sequencing were carried out for identification. The strains *Lactobacillus reuteri-E1* (*L. reuteri-E1*), *L. reuteri-E2*, and *L. reuteri-E3* were directly isolated from the feces of pigs that belonged to enterotype 1, 2, and 3, specifically. The *L. reuteri* was cultured using the MRS in the micro-anaerobic incubation system (LongYue, Shanghai).

### Microbiota Profiling

The 16S rDNA sequencing procedure was illustrated in detail in the Supplementary Method portion. And we briefly described the workflow here. The bacterial DNA was extracted by using a Stool DNA Kit (D4015-01, Omega Bio-tek, GA, America), then amplified with the V3–V4 region primers. The amplicons were purified, and then, sequenced *via* the Illumina MiSeq platform. The α diversity (Chao and Shannon index), β diversity (PCoA), microbial composition, differences between groups, and enterotype were analyzed after the assembly of the sequence. All raw sequencing data have been deposited in the National Center for Biotechnology Information (NCBI) Sequence Read Archive under the BioProject PRJNA793337.

## Results

### Native Chinese Breeds Show Stronger Diarrhea Resistance Than Commercial Hybrid Duroc-Landrace-Yorkshire Piglets

**Figure 1:**
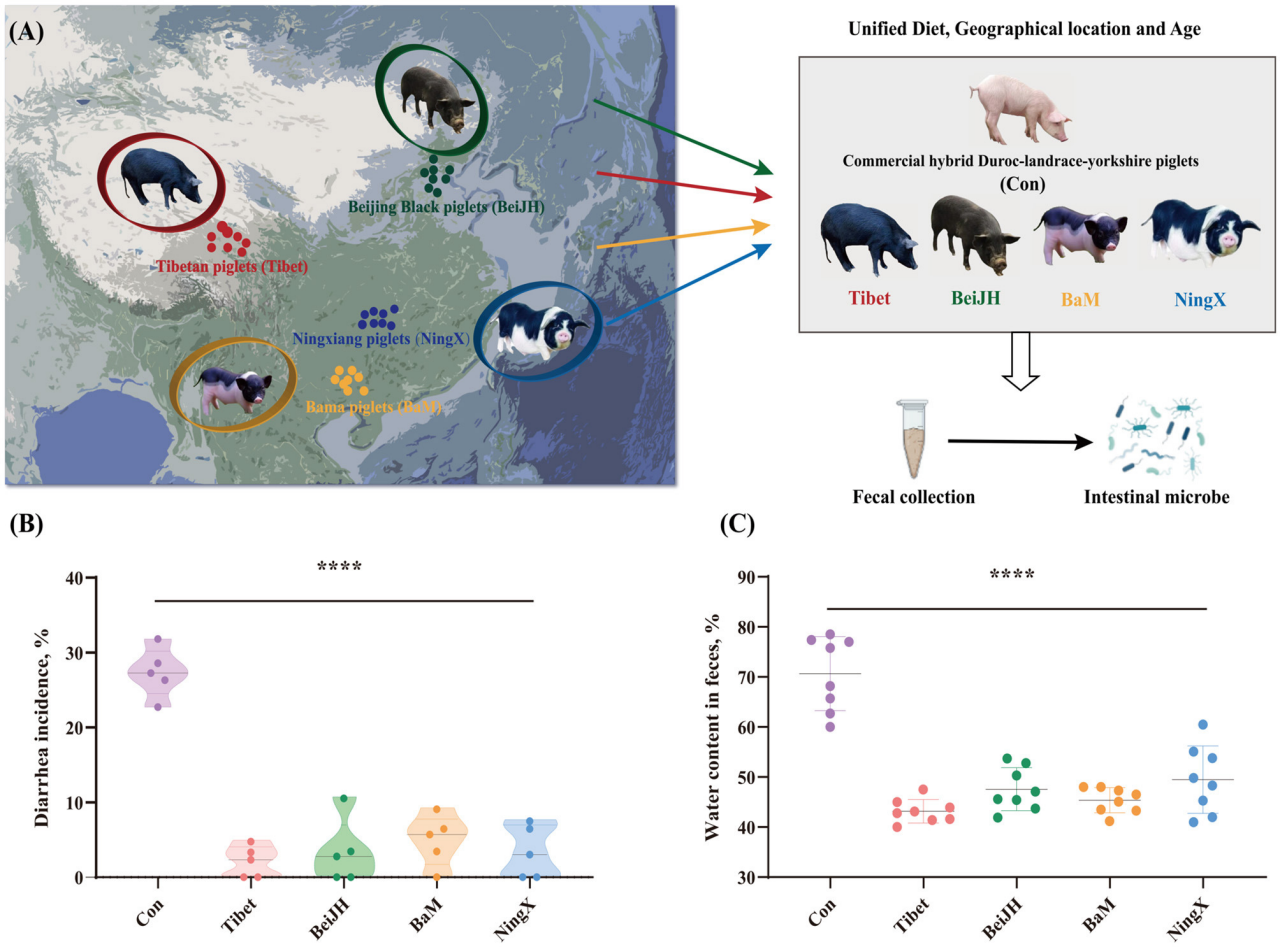
Native Chinese breeds showed stronger diarrhea resistance than commercial hybrid piglets. **(A)** The geographic location of the original population of four Chinese native pig breeds on the map, and the schematic diagram of the project process. **(B)** Chinese native pig breeds exhibited a significantly reduced diarrhea rate than commercial hybrid piglets. **(C)** Chinese native pig breeds showed reduced water content in feces. ****P < 0.05.

In pigs, various breeds have significantly variant anti-stress capacities, accompanied by discrepant gut microbiota. However, exploring the essential elements that shaped the differentiated microbe structure of some Chinese breeds is still challenging as are have widely different environmental factors from each other. To concentrate on the impact of genetic discrepancy in pig breeds and gut microbiota, exclusion of factors such as age, geographical location, and diet should be considered. So, in this study, some Chinese breeds, such as Tibet, BeiJH, BaM, and NingX, and the commercial hybrid Con, have been controlled to feed with the same diet (Supplementary Table 1), and in the same geographic location from their mother's generation (Figure 1A). In addition, the selected piglets among five breeds were of similar age after the weaning. Considering that the flora of piglets after weaning tends to be stable, this specific period can best reflect the true gut microbial composition among the variant pig breeds.

Fecal samples were collected on-site following a standardized procedure (see Supplementary Method). It can be found that the piglets of commercial hybrid are prone to diarrhea with a generally high diarrhea rate at about 27% (Figure 1B), which is accompanied by increased water content in feces (Figure 1C). In contrast, Chinese breeds all showed a visibly mild to even no diarrhea symptoms, which is consistent with a lower water content (Figures 1B,C).

This suggests that the intestinal microbiota co-evaluated with host genome. Genome is still the main mediator that affects the diarrhea rate and the intestinal health of the weaned piglets, even if external environmental factors are controlled.

### Various Breeds Have Significantly Variant Intestinal Micro-Environment

To profile the micro-coevolution of the intestinal microbiota among the Con and various Chinese breeds, we collected the bacteria of 40 piglets from 5 breeds using Illumina MiSeq sequencing of the V3–V4 region of bacterial 16S rRNA. After the size-filtering, quality control, and chimera removal, the total number of 3,905,171 sequences was obtained. Sequences were clustered into operational taxonomic units (OTUs) with 97% minimum identity. Additionally, the estimated sample coverage was more than 99.8% without exception, suggesting the reliable accuracy and the reproducibility of sequencing.

**Figure 2:**
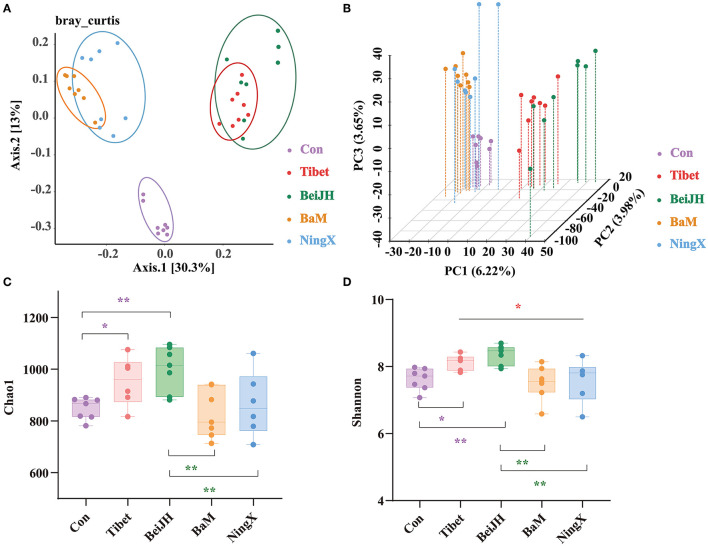
Species diversity analysis of microbial community. **(A)** Scatterplot from principal coordinates analysis (PCoA) in bacterial communities of fecal microbiota on genus level based on the bray_curtis distance. **(B)** 3D scatterplot from PCA in bacterial communities of fecal microbiota on genus level. The bacterial α diversity of fecal microbiota of piglets based on **(C)** Chao1 and **(D)** Shannon indexes. Data were shown as mean ± SD (n ≤ 8). Student's *T*-test was conducted. *P < 0.05; **P < 0.01.

The β-diversity was calculated at genus level. Results in principal coordinates analysis (PCoA), presented in the histogram, confirmed a significantly separate clustering between Con and Chinese breeds, with main principal component (PC) scores: PC1 = 30.3%, PC2 = 13%, demonstrating a different clustering (Figure 2A), which is consistent with Principal Component Analysis (PCA) analysis (Figure 2B) and with weighted unifrac-based PCoA (Supplementary Figure 1A). Meanwhile, the Tibet microbiota was similar to BeiJH microbiota, while the microbiota in BaM was similar to NingX. However, these two groups revealed a distinct clustering pattern, and between them, the Con group was more distributed (Figure 2A).

Chao1 and Shannon, two indicators describing the α diversity, were observed at the genus level. Tibet and BeiJH groups showed an obviously risen Chao1 and Shannon index compared to Con, while the α diversity of BaM and NingX significantly decreased compared with BeiJH, respectively (Figures 2C,D).

To evaluate the similarity in groups and to examine differences between treatments, a similarity analysis (ANOSIM/Adonis) was performed. Notably, differences between groups were greater than intragroup differences with the *P*-value of 0.001 (Supplementary Figure 1B). In addition, results of Partial Least Squares Discriminant Analysis (PLS-DA) also highlighted the similar microbiome composition in ethnically similar individuals (Supplementary Figure 1C), which collectively indicated the differential microbiota between various breeds.

### Characterizing the Gut Microbiota Between Various Pig Breeds

**Figure 3:**
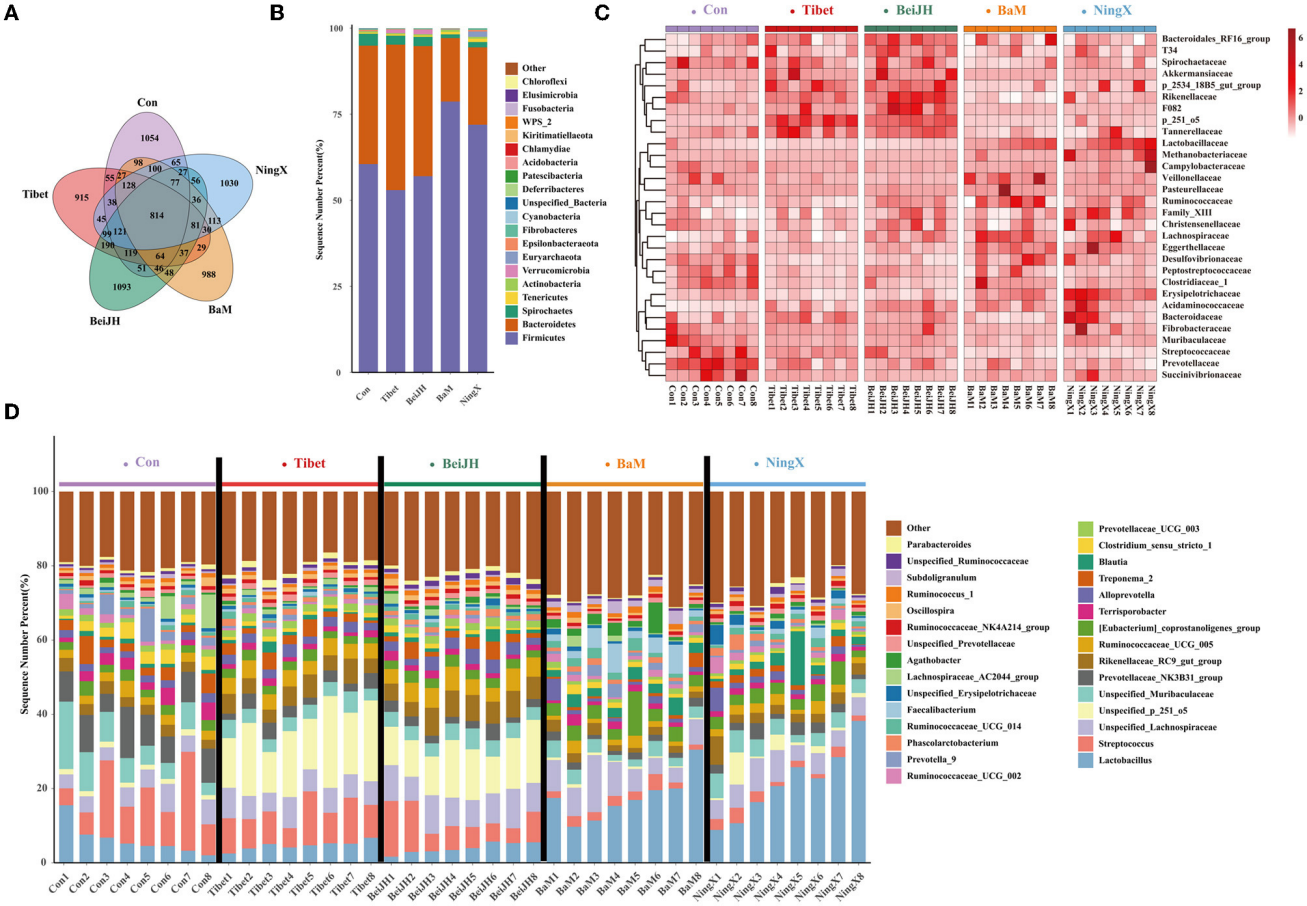
Differential composition of microbial community among various pig breeds. **(A)** Venn diagram analysis of five pig breeds. **(B)** Histogram of species abundance at the phylum level. **(C)** The heat map of bacteria composition at the family level. **(D)** Histogram of the microbial community at the genus level.

Sequences were clustered into 3275 OTUs, which were then binned into 393 genera, based on BLAST searches against the SILVA SSU database. Each breed had its own unique OTU category and has 814 OTUs in common (Figure 3A). A community bar-plot analysis at the phylum level exhibited a relative alteration of the microbial community. Firmicutes and Bacteroidetes were dominant phyla of feces. The Tibet and BeiJH possessed similar phylum structures with a decreased ratio of Firmicutes to Bacteroidetes; meanwhile, they also had a considerable proportion of Verrucomicrobia. Conversely, the consistent structure presented in BaM and NingX, in which, the Firmicutes proportion increased significantly, while the Bacteroidetes decreased significantly compared to the other three groups (Figure 3B). Relative abundances of microbes on family (Figure 3C) and genus (Figure 3D) levels were also presented in each breed. The internal microbiome taxonomic compositions between Tibet and BeiJH were relatively uniform, while the microbiome composition between BaM and NingX was also similar. However, their respective proportion and relative priority were all varied with Con, indicating that the differentiation between the microbiota and the host genetic admixture is consistent.

### Comparison of Unique Microbiota Profile Between Pig Breeds

**Figure 4:**
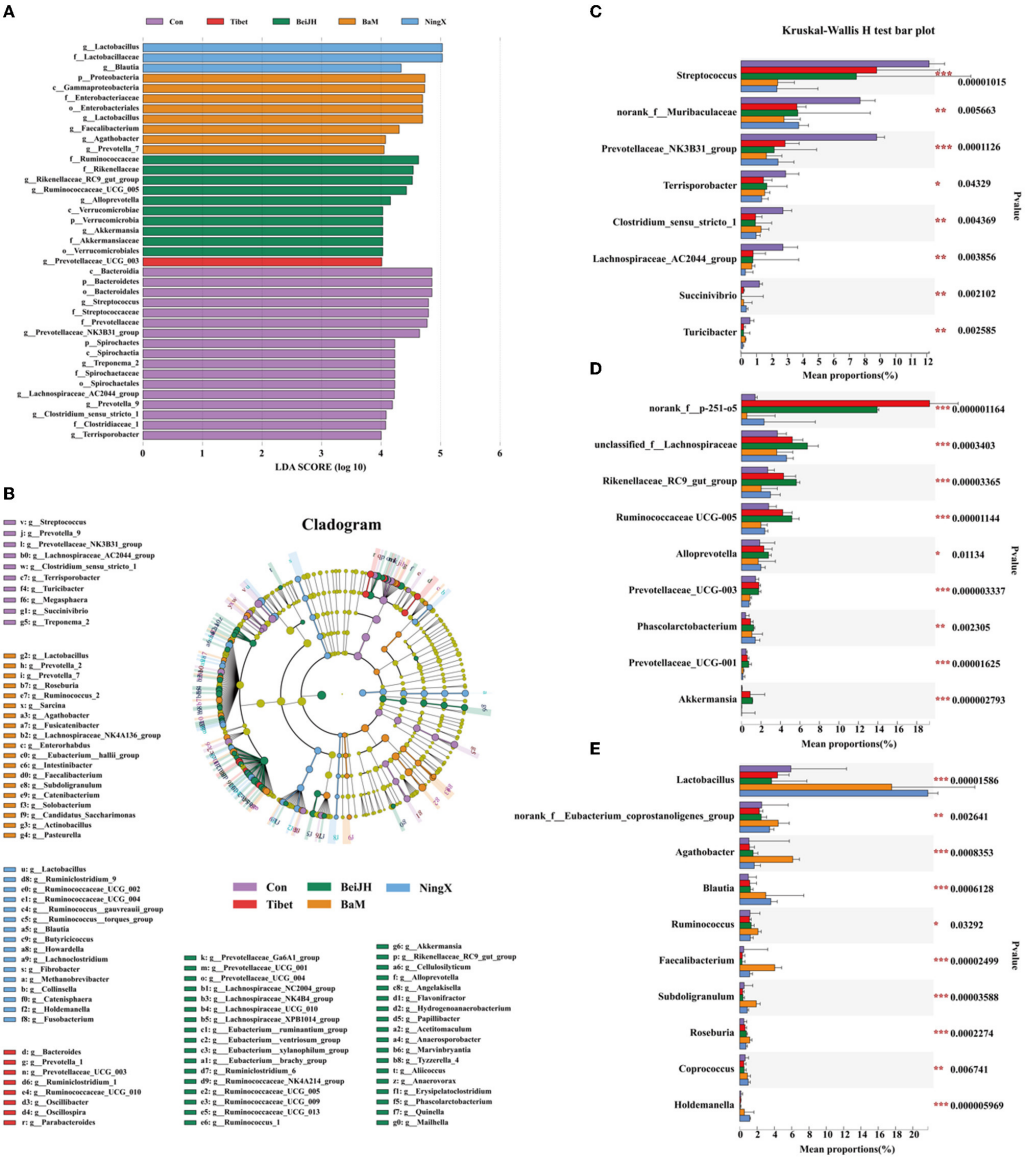
Various breeds had significantly variant intestinal micro-environment. Histogram **(A)** and cladogram **(B)** of Linear Discriminant Analysis (LDA) value distribution by setting score 4 at genus level in LEfSe analysis. **(C)** Kruskal–Wallis *H*-test bar plot of bacteria that were abundant in Con group. **(D)** Kruskal–Wallis *H*-test bar plot of bacteria that significantly increased in both Tibet and BeiJH groups. **(E)** Bacteria that gained increased proportion in BaM and NingX groups. *P < 0.05; **P < 0.01; ***P < 0.001.

In the comparison of the fecal microbiome by 16S rRNA gene amplicon sequencing analysis at genus level, we found that the fecal microbiota of Con was dominated by *Streptococcus, unspecified_Muribaculaceae, Prevotellaceae_NK3B31_group*, and *Terrisporobacter* (Figure 3D). Besides, *Lachnospiraceae_AC2044_group, Prevotella_9*, and *Clostridium_sensu_stricto_1* were also identified as the unique microbiota in Con (Figures 4A,B). The proportion of these bacteria was all significantly increased in the Con group (*P* < 0.05; Figure 4C).

Tibet and BeiJH were characterized by a similar microbiota profile, in which *Bacteroides_p_251_o5* possessed a considerable proportion (Figure 3D). According to LefSe analysis, BeiJH was also dominated by *Prevotellaceae_UCG_003*, while Tibet was also more abundant in *Rikenellaceae_RC9_gut_group, Ruminococcaceae_UCG_005, Alloprevotella*, and *Akkermansia* (Figures 4A,B). The Kruskal–Wallis *H*-test consistently showed higher levels of these mentioned bacteria (*P* < 0.05) in contrast with the other three pig breeds (Con, BaM, and NingX) (Figure 4D).

Relative to Con, indigenous pig breeds no matter in the south (represented by BaM and NingX) or living in the north (such as Tibet and BeiJH), both had a considerable proportion of *Lactobacillus* (Figure 3D). *Lactobacillus*, considered as a traditional prebiotic, was not only identified as the shared unique bacteria between BaM and NingX (Figures 4A,B) but has also displayed a significant increase compared to other three groups (Figure 4E). Other bacteria, associated with the generation of short-chain fatty acids (SCFAs), also had a higher ratio, including *Ruminococcus, Blautia, Faecalibacterium, Agathobacter*, and *Roseburia* (Figure 4E).

### Compositional Analysis of Enterotype-Like Clusters

**Figure 5:**
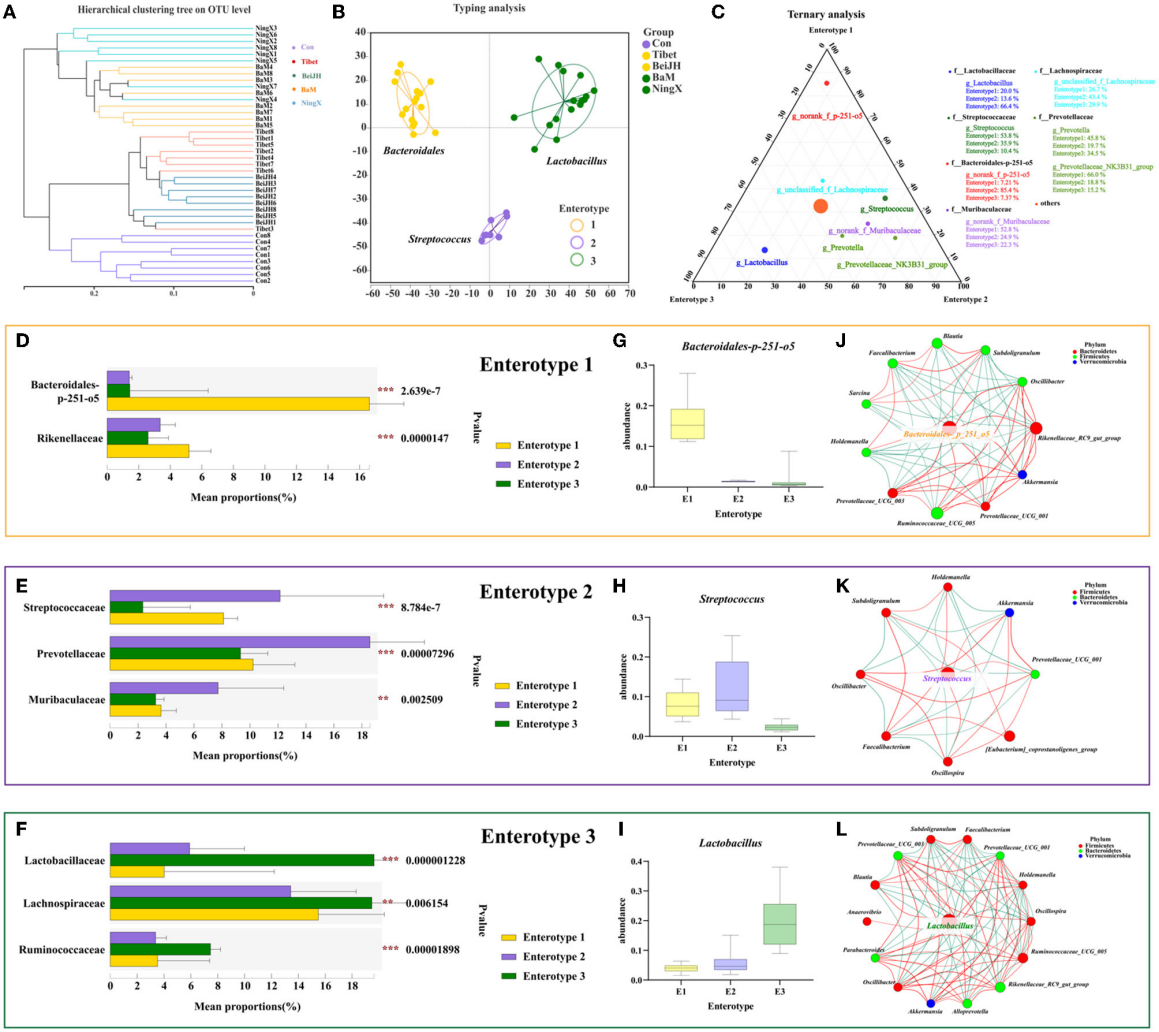
Compositional analysis of enterotype-like clusters. **(A)** Hierarchical clustering tree on OTU level. **(B)** The classification of enterotype among five pig breeds. **(C)** Ternary analysis for determinants in three enterotypes. Dominant bacteria in **(D)** Enterotype 1, **(E)** Enterotype 2, and **(F)** Enterotype 3. The abundance of *Bacteroidales-p-251-o5* **(G)**, *Streptococcus* **(H)**, and *Lactobacillus* **(I)**, in three enterotypes, specifically. **(J–L)** Enterotype characteristic bacteria were located in the center of each enterotype network interaction map. **P < 0.01; ***P < 0.001.

Microbial community structure seemed to reveal more subtle changes that were shaped by the host genetics (Figure 5A). BaM and NingX were hosts to the related *Lactobacillus*, while Tibet and BeiJH possessed specific clusters of *Bacteroides_p_251_o5*, and Con was dominated by *Streptococcus*. Accordingly, the enterotype clusters were subsequently classified based on broader-scale patterns across the host genetics and the geographical regions.

Microbiota communities were stratified into three robust enterotypes according to the previously described clustering method by Arumugam et al. (10) (Figure 5B, Supplementary Figure 2A). The driving genera were identified, which obtained the *Bacteroides* enterotype (E1), *Streptococcus* enterotype (E2), and *Lactobacillus* enterotype (E3) (Figures 5B,C). Among microbiome belonging to E2, Prevotellaceae still dominates in second place (Figure 5E). Meanwhie, in E3, Ruminococcus was also dominated (Figure 5F). Notably, Prevotellaceae and Ruminococcus are both enterobacteriaceae that identified in human enterotype previously (11). Consistent with the classification of human enterotypes, the enterotype of piglets had also obtained three significant clusters based on their common and their characteristic bacteria (Supplementary Figure 2B). Moreover, the enterotype-associated host genetics was also reflected in the differentiation of microbiome genetics (Supplementary Figure 2C), collectively illustrating the micro-coevolution of host and microbes.

It can be found that the gradient distribution of the genera *Bacteroides-p251-o5* (Figures 5D,G,J), *Streptococcus* (Figures 5E,H), and *Lactobacillus* (Figures 5F,I) was highly correlated with enterotype clustering. Additionally, the best driver of each enterotype was identified by the network of the co-occurring genera, which centered around its specific driving genus. It can be consistently found that since E1 was driven by *Bacteroides* (Figure 5J), *Streptococcus* was the most dominant contributor causing differences between groups and the similarity within E2 (Figure 5K), while the best driver for E3 was *Lactobacillus* (Figure 5L).

### Screening and Function Exploration of the E3 Representative Strain *Lactobacillus reuteri* Among the Three Other Enterotypes

**Figure 6:**
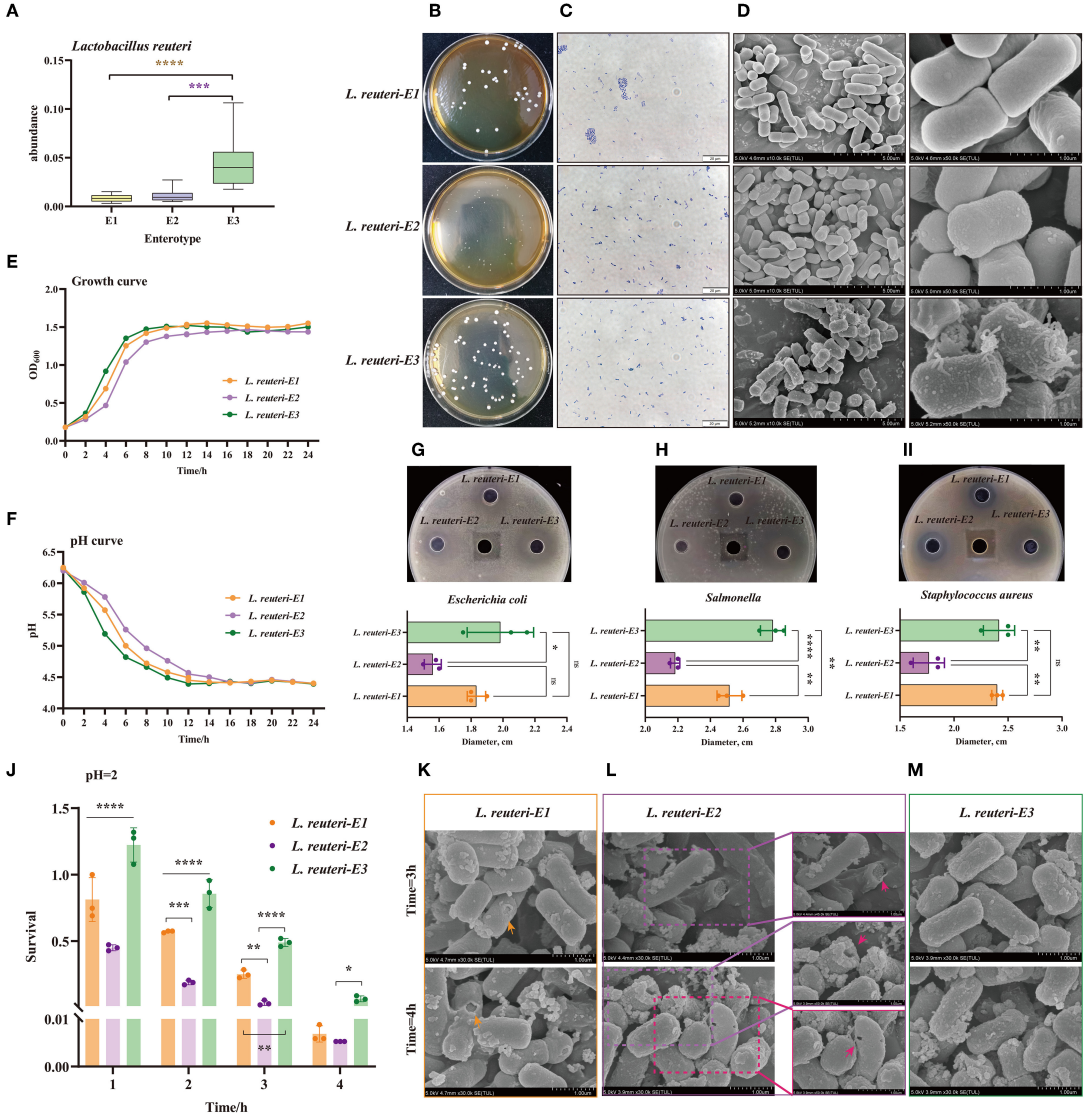
Screening and function exploration of E3 representative strain *Lactobacillus reuteri* (*L. reuteri*) among three enterotypes. **(A)** The abundance of *L. reuteri* in three Enterotypes. **(B)** Photograph of the plate, **(C)** Gram stain, and **(D)** SEM pictures of *L. reuteri* isolated from the three enterotypes. The magnification is 10.0k. **(E)** Growth curve analysis of three strains of *L. reuteri*. **(F)** Comparison of the acid production capacity of three strains of *L. reuteri*. The inhibition against **(G)** *Escherichia coli*, **(H)** *Salmonella*, and **(I)** *Staphylococcus aureus*. **(J)** Comparison of the survival rates of the three types of *L. reuteri* under the condition of pH = 2. Scanning electron microscope (SEM) picture of the destruction of the outer membrane of **(K)** *L. reuteri*-E1, **(L)** *L. reuteri*-E1, and **(M)** *L. reuteri*-E3. *P < 0.05; **P < 0.01; ***P < 0.001; ****P < 0.0001.

Distinct enterotypes corresponded to different functional annotations of gut microbiota. Among them, the SCFA and lactic acid-production-related enterotype (E3) was expected to be healthier. *L. reuteri* was the characteristic bacteria of E3, which was significantly different from the other two enterotypes (Figure 6A). Based on enrichment culture and screening of acids production, we have successfully isolated and identified three desired strains of *L. reuteri*, which were isolated from E1, E2, and E3, and, were named was *L. reuteri-E1, E2*, and *E3*, accordingly (Figure 6C, Supplementary Figure 3). The isolated strain is white, opaque, with neat edges, and smooth and round convex surfaces. It can be found that the colony of *L. reuteri-E3* is thicker, while the *L. reuteri-E2*'s is the smallest and thinnest (Figure 6B). Scanning electron microscopy (SEM) results also show that filamentous protrusions are formed on the surface of *L. reuteri-E3*, which is related to the secretion of polysaccharides (Figure 6D). The *L. reuteri-E3* has a superior growth rate than the *L. reuteri-E1* and *E2*, in which it started the logarithmic growth phase at 4 h and the plateau stage at 8 h (Figure 6E). The *L. reuteri-E3* has also a desirable capacity of producing acid, which pH value dropped to 4.39 after 12 h of cultivation (Figure 6F), concurrent with the inhibition against *Escherichia coli* (Figure 6G), *Salmonella* (Figure 6H), and *Staphylococcus aureus* (Figure 6I). In addition, the *L. Reuteri-E3* has the strongest ability to withstand low pH values, which is prominently manifested in the integrity of the bacterial membrane (Figure 6M) and the maintenance of a higher survival rate (Figure 6J, Supplementary Figure 4). However, although *L. reuteri-E2* shows better performance than *L. reuteri-E1* (Figure 6J), there are still pores in the bacterial membrane under an extremely acidic condition (Figures 6K,L).

## Discussion

Based on the characteristic of low diarrhea in the native Chinese breeds, we introduced an enterotype model for piglets, which is a new perspective to decipher the colonization and the transition of the gut microbiota among various pig breeds (15). After removing environmental effects on the gut microbiome, including geographical location and diet, the function of different genetic backgrounds of pig breeds on enterotype clusters was mainly focused, since the microbiota shaped by host genetics is much more deterministic and predictable. Three representative enterotype clusters were identified, which were represented by *Bacteroides, Streptococcus*, and *Lactobacillus*. Native Chinese breeds were distributed in E1 and E3, which collectively drove the diversification and functionality of the microbial community of various Chinese pig breeds. Furthermore, the *L. reuteri*, which is the representative strain of E3, was specifically isolated among three enterotypes. The excellent stress-resistance of *L. reuteri-E3* not only highlights the stronger disease resistance of Chinese breeds but also constructs the picture of the micro-coevolution of the human host genetics with the gut microbiome.

China possesses abundant native pig resources, some of which always show stronger disease resistance in practical production, such as the Tibet pigs, Bama pigs, and so on. Various breeds have significantly variant anti-stress capacities and accompany a discrepant gut microbiota (1, 2). Gut microbiota contributes to the disease resistance, which is partially regulated by their genetic background.

As one of the Chinese native breeds, Tibet pigs are strong in anti-stress capacity, characterized by a lower diarrhea rate (16). The BeiJH pigs are derived from the local North China pigs in Beijing, with a characteristic tolerance to rough-feeding and anti-stress. Bama pigs serve as ideal models for biomedical research, which are exhibited by a high intramuscular fat content (17). In addition, NingX pigs, a well-known Chinese indigenous fatty-type breed, have gained more diverse bacterial communities (18). Although the performance, appearance, and original geographic location of these breeds are variant, the microbiota does contribute to shaping their anti-stress ability compared to commercial hybrid piglets.

It is well-acknowledged that diet and geography can alter gut microbial composition and metabolism (19). To eliminate the impact of environmental factors (geographical location and diet) that are likely to cause microorganism turbulence as much as possible, four types of native Chinese breeds are removed from the same place with commercial hybrid Duroc-landrace-Yorkshire piglets, while feeding with the same diet. Here, the composition of the microbiome differed in the feces among the five breeds. Tibet and BeiJH pigs possess a similar microbial structure; the microbial evolution of BaM and NingX is closer. However, they are all very different from commercial hybrid pigs.

Following the designation of enterotypes in humans (10) and chimps (20), the driver bacteria of E1 in our study is Bacteroides, which is coincident in humans and chimps. Contributors to “E2 and E3” in piglets follow a similar pattern with humans, however, their arrangement within the enterotype has changed. Specifically, the order of *Prevotella* (a significant contributor to human E2) drops after the *Streptococcus* in E2 in pigs, resulting from an overall reduction of *Prevotella* abundance in pigs than in humans (13). In addition, the *Ruminococcus*-dominated enterotype in humans is replaced by *Lactobacillus*, which is the major contributing genus to E3 in pigs. Interestingly, major contributors in human enterotypes are all present in the enterotype core clusters in pigs, suggesting the homologic similarity between pigs and humans. However, *Streptococcus* and *Lactobacillus* in pigs are both absent in the human enterotype (21), suggesting that pig communities may lack some vital biological functions.

The *Bacteroides* enterotype is dominant in Tibet and BeiJH groups, suggesting a stronger degradation ability for carbohydrates than for E2 and E3 (22). The close relation between *Bacteroides*/*Prevotella* indicates that the preferred utilization of fiber or protein has been widely shown (11). Other remarkable genus in E1 also include *Rikenellaceae_RC9_gut_groups, Lachnospiraceae, Ruminococcaceae UCG-005*, and *Anaerovorax* (17), which are known for the ability to ferment dietary polysaccharides or plant fibers. *Akkermansia* also showed a remarkable predominance in E1. *Akkermansia* is an intestinal symbiont colonizing in the mucosal layer, which is considered to be a promising candidate as a probiotic. *Akkermansia* is known to have an important value in improving the host's metabolic functions and immune responses, also improving host defense in mild inflammatory conditions, and increasing mucus production by promoting the differentiation of secretory intestinal epithelial cells lineages (23). Moreover, members of Lachnospiraceae, Rikenellaceae, and Bacteroidaceae families are also identified to compete with pathogens for mucin-derived sugars and, therefore, serve as ecological gatekeepers in healthy guts (24). The structure of E1 is consistent with the original harsh natural conditions for Tibet and BeiJH to survive, and their instinct to digest a high-fiber diet. The main representative strain of E2 is *Streptococcus*, while the bacteria, such as *Succinivibrio, Terrisporobacter*, and Muribaculaceae, were also significantly increased in E2. The *Streptococcus* includes groups A and B: group A Streptococcus (GAS) species are responsible for a wide variety of human diseases that range from noninvasive, mild infections, to life-threatening, and invasive conditions (25); and Group B *Streptococcus* (GBS) remains a leading cause of serious neonatal infection (26). Moreover, the functional coverage of *Terrisporobacter* is controversial. Sometimes, it is considered as pathogenic bacteria (27) that is linked to a polymicrobial infection in clinical cases (28). However, the genus *Terrisporobacter* is also closely related to SCFA contents and oxidative indicators (29) and plays a key role in degrading organic matter (28). The *Succinivibrio* was also enriched in E2, which is regarded as a double-edged sword, not only involved in the accumulation of anti-inflammatory cytokines and viral inhibitors but also induced the inflammatory responses in the gut (30). Besides being converted into propionate by the cross-feeding effect among the microbiota, *Succinivibrio* is also metabolized to directly succinate, which could activate immune cells and aggravate inflammation (31). Muribaculaceae was also obviously increased in E2, which is functionally distinct from the neighboring families and is versatile concerning the complex carbohydrate degradation (32), especially for mucin degradation (33). Thus, it is considered a major mucin monosaccharide forager (24). Additionally, the abundant microbes that generate postbiotics are gathered in the E3, including lactic acid-producing bacteria, *Lactobacillus*, butyric acid-generating bacteria, *Blautia* (34), *Roseburia* (35), and *Holdemanella* (36).

We further isolated the dominant strain *L. reuteri* from E3 and separated its equivalents, *L. reuteri-E1* and *L. reuteri-E2*, to explore their different structure and function associated with host genetics. We found that *L. reuteri* from E3 has stronger resistance to stress, and the growth rate, acid production capacity, and antibacterial properties of *L. reuteri-E3* are also more advantageous. Thus, unitizing the micro-coevolution of intestinal microbiome and native Chinese breeds is warranted to improve anti-diarrhea treatment strategies.

Our study provides several points of enterotype-like community clusters in pigs. However, larger populations, more breeds, and comprehensive physiological indices are still required to divide a more deterministic and predictable enterotype in pigs. In addition, the effect of covariates and a wider survey of nutritional sources on the gut microbiota is warranted to accurately assess the composition of enterotypes and possible transformation factors.

In conclusion, we put forward three functionally enterotype-like clusters present in Chinese native breeds and commercial hybrid pigs. Remarkably, enterotypes in pigs display some common characteristics with humans and chimpanzees as reported, indicating the existence of ancient shared traits in mammalian hosts. However, some unique contributors in pig enterotype emphasize the micro-coevolution of intestinal microbiota and pig genetics. Although controlling environmental effects relatively well, the host genetic still essentially contributes to enterotype status, which is essential for the deterministic and predictable regulation of microbiota. Moreover, some remarkable genus, as well as their generating post-biotics within specific enterotypes, may also have great potential as biomarkers for intervening the weaned piglet diseases, thus, relying on diversified Chinese native pig resources with strong stress resistance.

## Data Availability Statement

The datasets supporting the conclusions of this article are available in the NCBI Sequence Read Archive (SRA) repository under accession number PRJNA793337.

## Ethics Statement

The animal study was reviewed and approved by the Animal Care and Use Ethics Committee of China Agricultural University.

## Author Contributions

XM conceived and designed the study, gave a critical reading and editing, and resourced the project. NM conducted most of the *in vitro* bacteria experiments, performed the statistical analysis for all the data, and wrote the original draft. JC contributed to collecting fecal samples. YS, ZQ, and CL performed Gram staining, inhibition zone determination, and acid resistance-related experiments. All authors read and approved the final manuscript.

## Funding

This work was supported by the National Natural Science Foundation of China (31930106 and 31829004), the National Ten-thousand Talents Program of China (23070201), the Henan Province Public Benefit Research Foundation (201300111200-05), the 2115 Talent Development Program of China Agricultural University (1041-00109019), and the 111 Project (B16044).

## Conflict of Interest

The authors declare that the research was conducted in the absence of any commercial or financial relationships that could be construed as a potential conflict of interest.

## Publisher's Note

All claims expressed in this article are solely those of the authors and do not necessarily represent those of their affiliated organizations, or those of the publisher, the editors and the reviewers. Any product that may be evaluated in this article, or claim that may be made by its manufacturer, is not guaranteed or endorsed by the publisher.
