# Supplementary material for: Micro-Coevolution of Genetics Rather Than Diet With Enterotype in Pigs
Source: Front Nutr. 2022 Mar 22;9:846974. doi: 10.3389/fnut.2022.846974 (PMC8982514; doi:10.3389/fnut.2022.846974)
Supplement: Supplementary file 1 [file Data_Sheet_1.docx]

Supplementary Material

Micro-Coevolution of Genetics Rather than Diet with Enterotype in Pigs

# Supplementary Method Details

## Sample Collection

Fecal samples were collected following a standardized procedure: use centrifuge tubes to collect fresh feces of different pig breeds, while ensuring that the feces will not fall on the ground or be contaminated by other substances during collection. samples were transported to the freezer at sampling site within 0.5 hour, and further to the research laboratory with cold-chain within 1 day; samples were then well homogenized, aliquoted, and stored at−80 °C until further analyses.

## 16S rDNA Sequencing and Data Analysis

Microbial genomic DNA from feces was all extracted by commercial OMEGA Stool DNA Kits. Genomic DNA was amplified using specific primers with the barcode locked in 16S V3-V4 region. Paired-end sequencings were read on the Illumina MiSeq platform. An OTU table was obtained from the Mothur Bayesian classifier. The original sequencing data were filtered and processed to get the valid data, in which DADA2 method recommended by QIIME2 was applied to de-noise, merge and de-chimera. Based on valid data, OTU clustering/denoising and species classification analysis were performed to form the species abundance spectrum of OTU and other species classification grades after data homogenization. Then, the abundance and diversity index of OTU were analyzed, and the community structure of species annotation referring to Silver database was statistically analyzed at each taxonomic level. Principal coordinate analysis (PCoA) was performed to get principal coordinates and visualize from intricate data. Shannon and Chao1 indexes are used to evaluate the complexity of species diversity.

## Identification of Enterotypes

For 16S sequencing data, the relative taxonomic abundances at the OTU and genus levels were used as taxonomic features for enterotype identification. First, the JSD at the genus and pathway levels and the unweighted/weighted UniFrac distances at the OTU level between each sample were calculated to produce distance matrices. Second, the partitioning around medoids (PAM) clustering algorithm was performed on these distance matrices to cluster samples, using the Calinski-Harabasz (CH) index to assess the optimal number of clusters. Third, the silhouette index (SI) was calculated to evaluate the statistical significance of clustering at each number of clusters. The PAM clustering algorithm and tools to calculate the CH index and the SI are available in the R packages ‘cluster’ and ‘clusterSim’.

## Determination of Fecal Water

Fresh stools were immediately collected into 1.5 mL eppendorf tubes and sealed on ice. After weighing, stools placed into the pre-tared weighing disks were completely desiccated by incubation in a dry oven at 105 °C for 12 h, and then were cooled in dryer and weighed until the difference of two successive weighing is less than 0.002. Fecal water was quantified as the ratio of mass loss of samples upon desiccation.

# Supplementary Figures


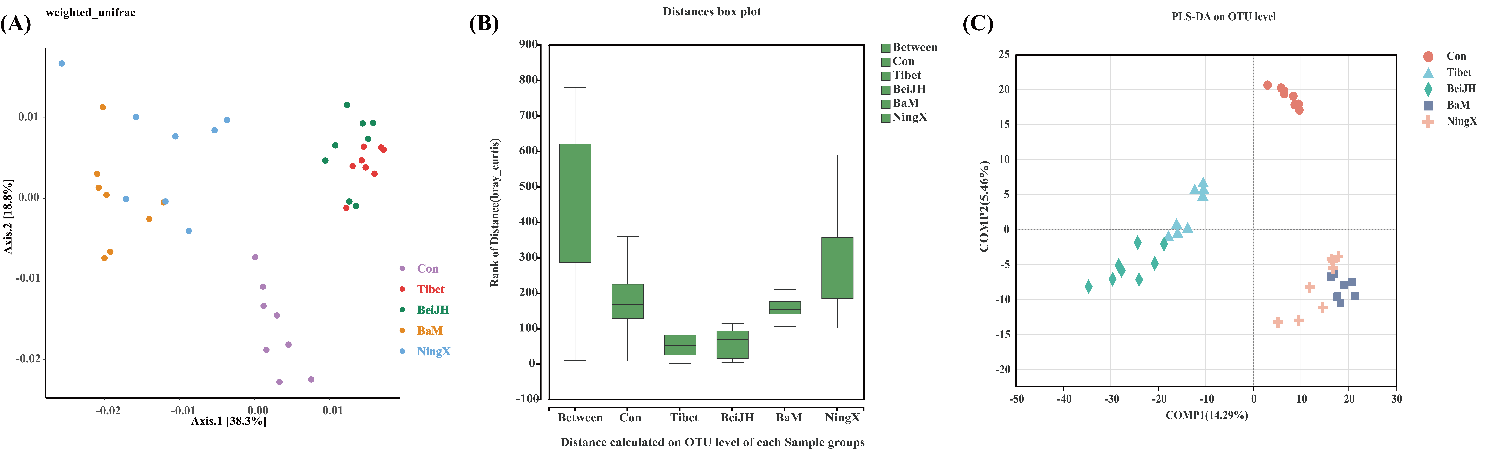


Supplementary Figure 1. Diversity analysis of bacterial composition. (A) Scatterplot from PCoA in bacterial communities of fecal microbiota on genus level based on the weighted_unifrac distance. (B) Distance calculated on OTU level of each Sample groups. (C) PLS-DA on OTU level.

**
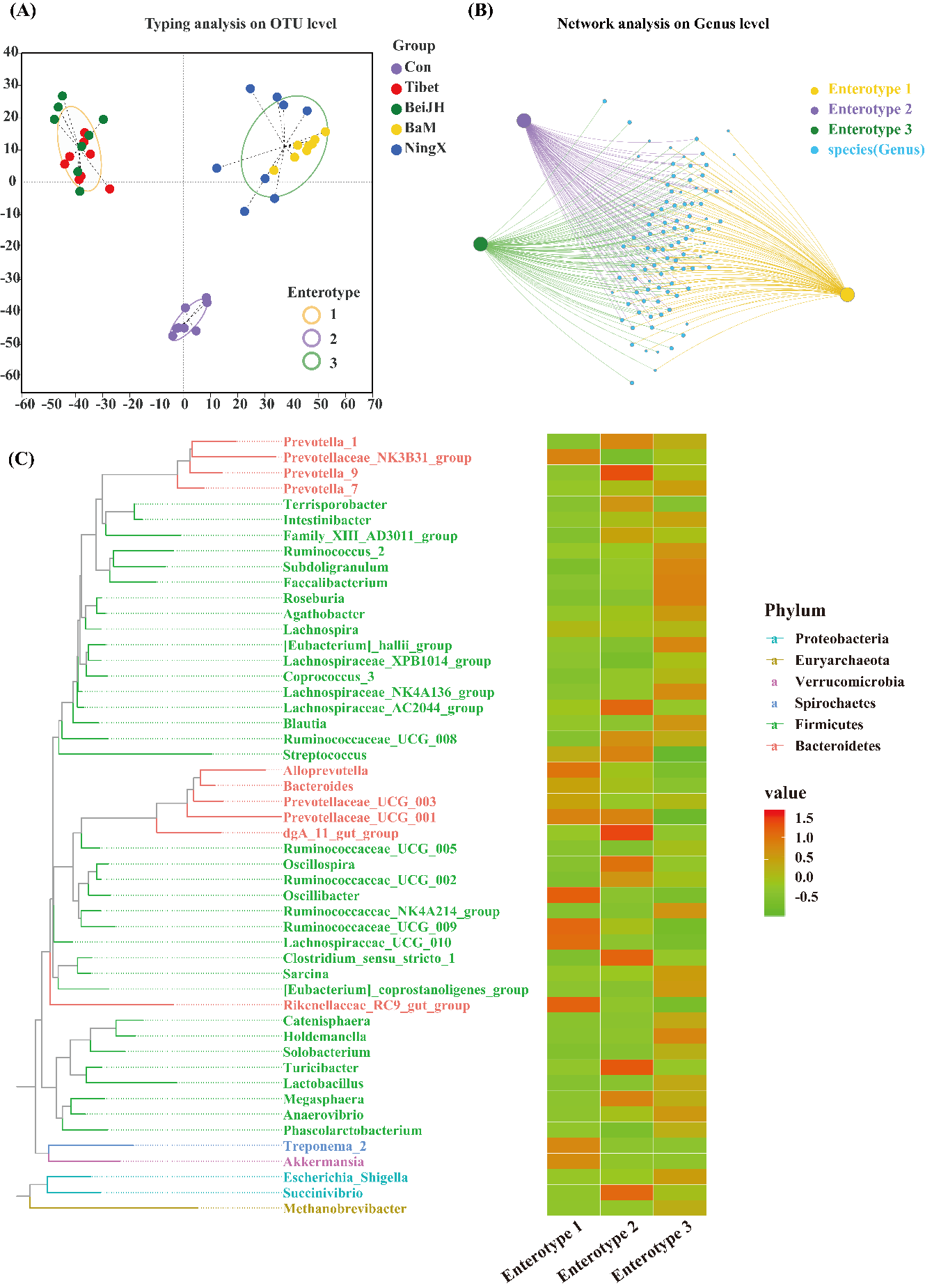
**

**Supplementary Figure 2.** **Intestinal typing and species evolution analysis.** **(A)** The classification of enterotype among five pig breeds. Colors are distinguished by pig breeds. **(B)** Network analysis on genus level of three enterotypes. **(C)** The evolutionary relationship of bacteria contained in the three enterotypes.

**
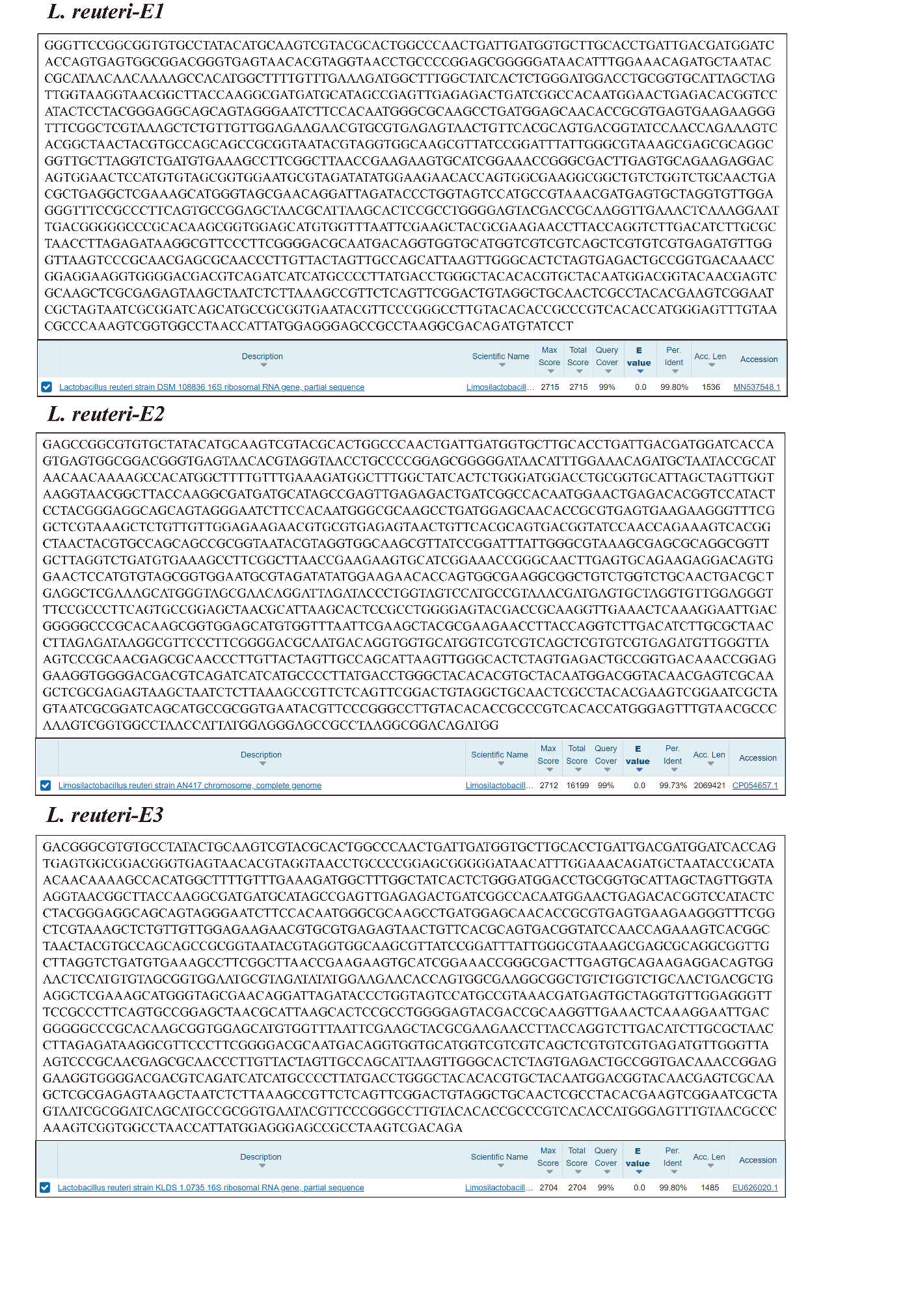
**

**Supplementary Figure 3.** **The 16s rDNA sequence of three stains of *Lactobacillus reuteri* and the result of strain BLAST on NCBI.**

**
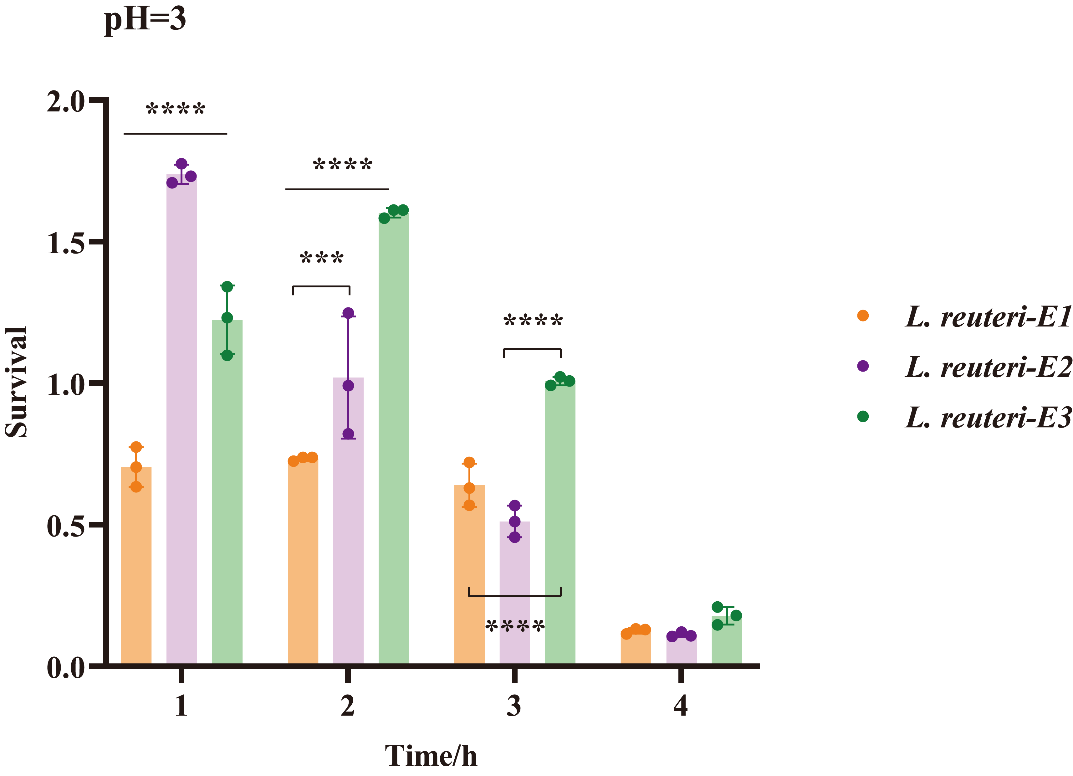
**

**Supplementary Figure 4. Comparison of the survival rates of the three types of *Lactobacillus reuter*i under the condition of pH=3.**

**Supplementary Table 1** The ingredient and nutrient content of the basal diets (dry matter basis, %)

| Ingredients | Content (%) | Nutrient levels | Content (%) |
| --- | --- | --- | --- |
| Corn | 63.25 | DE, MJ/kg | 14.64 |
| Soybean | 15.5 | CP, % | 18.1 |
| Puffed full-fat soybeans | 8 | Lysine, % | 1.38 |
| Fish meal (64.6%) | 4 | Met, % | 0.41 |
| Whey powder (3.8%) | 4 | Thr, % | 0.85 |
| Soybean oil | 2.27 | Trp, % | 0.24 |
| Dicalcium phosphate | 0.66 | Ca, % | 0.7 |
| Limestone | 0.8 | TP, % | 0.54 |
| Salt | 0.3 | AP, % | 0.33 |
| L-lys | 0.45 |  |  |
| DL-met | 0.08 |  |  |
| Thr | 0.15 |  |  |
| Trp | 0.04 |  |  |
| Premix^b^ | 0.5 |  |  |
| Total | 100 |  |  |

*Premix provided the following per kg of diet: vitamin A, 12,000 IU; vitamin D3, 3000 IU; vitamin E, 30 IU; vitamin K3, 2.5 mg; vitamin B12,20.0 µg; riboflavin, 4.0 mg; pantothenic acid, 12.5 mg; niacin, 40 mg; choline chloride, 400 mg; folacin, 0.7 mg; thiamine 2.5 mg; pyridoxine 3.0 mg; biotin, 70 µg; Mn, 30 mg; Fe, 100 mg; Zn, 80 mg (ZnO); Cu, 90 mg; I, 0.25 mg; Se, 0.15 mg. DE, digestible energy; CP, crude protein; Met, methionine; Thr, threonine; Trp, tryptophan; Ca, calcium; TP, total phosphorus; AP, available phosphorus.*
